# Identifying a Birth Cohort of Twins from Linked Data – Challenges and Opportunities

**DOI:** 10.23889/ijpds.v9i5.2768

**Published:** 2024-09-18

**Authors:** Alexander Charles Campbell, Jesse T Young

**Affiliations:** 1 Centre for Epidemiology and Biostatistics, Melbourne School of Population and Global Health, The University of Melbourne; 2 Centre for Adolescent Health, Murdoch Children’s Research Institute; 3 Justice Health Group, Curtin School of Population Health, Curtin University; 4 Institute for Mental Health Policy Research, Centre for Addiction and Mental Health; 5 Dalla Lana School of Public Health, University of Toronto

## Objective

In the absence of accurate perinatal records, identifying and differentiating twins in administrative records is difficult because of their similarity; they share surnames, dates of birth, and residences. In administrative databases, events within twin pairs are often incorrectly identified as being from one individual, representing a threat to data accuracy. An inability to identify twins in administrative data precludes applying powerful twin-based causal inference methods to understand health and social well-being.

## Approach

We developed an algorithm using linked administrative birth, perinatal, emergency department, and hospital records in Victoria, Australia from 1 January 1993 to 31 December 2023. We probabilistically linked a sample of 1,434 ‘known’ twin pairs from the Australian Twin Registry to validate the sensitivity of our algorithm. We calculated specificity using a sample of non-twins derived from linked data.

## Results

Our algorithm identified 37,900 twin pairs, 75,800 twin individuals, in the Victorian linked dataset. The accuracy of our ascertainment of twins by key characteristics will be presented and discussed.

## Conclusions

The birth cohort we have generated is one of the largest twin cohorts in the world with unprecedented granularity in longitudinal health and social data.

## Implications

Our algorithm improves the accuracy of administrative data repositories and linkage, providing novel information on where errors in previous linkages have occurred due to limited familial or intergenerational information. Our large population-based birth cohort of twins can be used to investigate the familial and non-familial determinants of health using advanced causal inference which leverages the natural similarity between twins.

